# *cis*-Aqua­bis­(2,2′-bipyrimidine-κ^2^ *N* ^1^,*N* ^1′^)iodidomanganese(II) iodide dihydrate

**DOI:** 10.1107/S1600536811037810

**Published:** 2011-09-30

**Authors:** Kwang Ha

**Affiliations:** a School of Applied Chemical Engineering, The Research Institute of Catalysis, Chonnam National University, Gwangju 500-757, Republic of Korea

## Abstract

The asymmetric unit of the title compound, [MnI(C_8_H_6_N_4_)_2_(H_2_O)]I·2H_2_O, contains a cationic Mn^II^ complex, an I^−^ anion and two solvent water mol­ecules. In the complex, the Mn^II^ ion is six-coordinated in a considerably distorted octa­hedral environment defined by four N atoms of the two chelating 2,2′-bipyrimidine (bpym) ligands, one I^−^ anion and one O atom of a water ligand. As a result of the different *trans* effects of the I and O atoms, the Mn—N bond *trans* to the I atom is slightly longer than the Mn—N bond *trans* to the O atom. The dihedral angle between the least-squares planes of the two bpym ligands [maximum deviation = 0.088 (4) Å] is 76.48 (6)°. In the crystal, the complex cation, the anion and the solvent water mol­ecules are linked by inter­molecular O—H⋯O, O—H⋯I and O—H⋯N hydrogen bonds.

## Related literature

For the crystal structures of mononuclear 2,2′-bipyrimidine Mn^II^ complexes, see: Hong *et al.* (1996 ▶); Smith *et al.* (2001 ▶); Ha (2011 ▶).
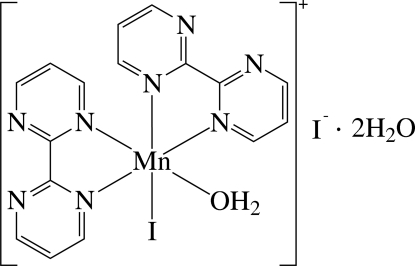


## Experimental

### 

#### Crystal data

[MnI(C_8_H_6_N_4_)_2_(H_2_O)]I·2H_2_O
*M* *_r_* = 679.12
Monoclinic, 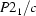

*a* = 14.2105 (12) Å
*b* = 21.5452 (19) Å
*c* = 7.7064 (7) Å
β = 102.063 (2)°
*V* = 2307.4 (4) Å^3^
*Z* = 4
Mo *K*α radiation
μ = 3.28 mm^−1^
*T* = 200 K
0.25 × 0.23 × 0.11 mm

#### Data collection

Bruker SMART 1000 CCD diffractometer
Absorption correction: multi-scan (*SADABS*; Bruker, 2000 ▶) *T*_min_ = 0.838, *T*_max_ = 1.000
17004 measured reflections
5707 independent reflections
3555 reflections with *I* > 2σ(*I*)
*R*_int_ = 0.049

#### Refinement

*R*[*F* ^2^ > 2σ(*F* ^2^)] = 0.037
*wR*(*F* ^2^) = 0.096
*S* = 1.05
5707 reflections
271 parameters
H-atom parameters constrained
Δρ_max_ = 0.97 e Å^−3^
Δρ_min_ = −1.15 e Å^−3^

### 

Data collection: *SMART* (Bruker, 2000 ▶); cell refinement: *SAINT* (Bruker, 2000 ▶); data reduction: *SAINT*; program(s) used to solve structure: *SHELXS97* (Sheldrick, 2008 ▶); program(s) used to refine structure: *SHELXL97* (Sheldrick, 2008 ▶); molecular graphics: *ORTEP-3* (Farrugia, 1997 ▶) and *PLATON* (Spek, 2009 ▶); software used to prepare material for publication: *SHELXL97*.

## Supplementary Material

Crystal structure: contains datablock(s) global, I. DOI: 10.1107/S1600536811037810/aa2026sup1.cif

Structure factors: contains datablock(s) I. DOI: 10.1107/S1600536811037810/aa2026Isup2.hkl

Additional supplementary materials:  crystallographic information; 3D view; checkCIF report

## Figures and Tables

**Table:** 

Mn1—O1	2.131 (3)
Mn1—N1	2.253 (4)
Mn1—N4	2.266 (4)
Mn1—N5	2.270 (4)
Mn1—N8	2.310 (4)
Mn1—I1	2.8070 (8)

**Table:** 

N1—Mn1—N4	72.96 (13)
N5—Mn1—N8	72.47 (13)

**Table 2:** Hydrogen-bond geometry (Å, °)

*D*—H⋯*A*	*D*—H	H⋯*A*	*D*⋯*A*	*D*—H⋯*A*
O1—H1*A*⋯O2	0.84	1.93	2.753 (4)	166
O1—H1*B*⋯O2^i^	0.84	1.87	2.693 (4)	166
O2—H2*A*⋯I2	0.84	2.63	3.419 (3)	157
O2—H2*B*⋯N6^ii^	0.84	2.16	2.948 (5)	157
O2—H2*B*⋯N7^ii^	0.84	2.29	2.884 (5)	128
O3—H3*A*⋯I2	0.84	2.82	3.624 (4)	161
O3—H3*B*⋯I2^iii^	0.84	2.73	3.517 (4)	157

Symmetry codes: (i) 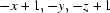; (ii) 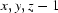; (iii) 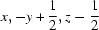.

**Fig. 1.**
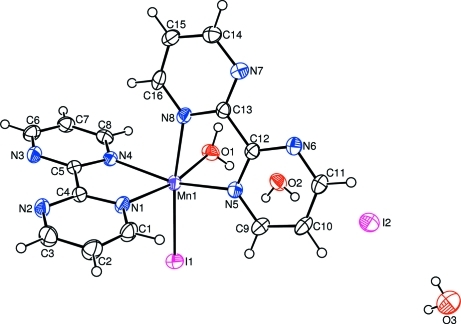
The structure of the title compound, with displacement ellipsoids drawn at the 40% probability level for non-H atoms.

**Fig. 2.**
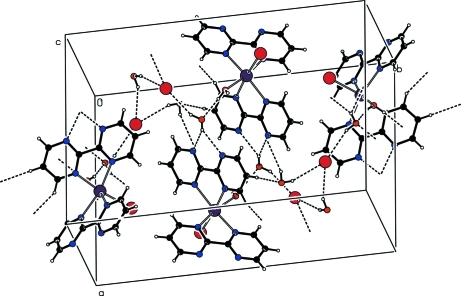
View of the unit-cell contents of the title compound. Hydrogen-bond interactions are drawn with dashed lines.

## supplementary crystallographic information

### Comment

Mononuclear Mn^II^ complexes of 2,2'-bipyrimidine (*bpym*, C_8_H_6_N_4_) ligand, such as [Mn(*bpym*)_2_(H_2_O)_2_](ClO_4_)_2_^.^2H_2_O (Hong *et al.*, 1996), [Mn(*bpym*)_2_(H_2_O)_2_](BF_4_)_2_^.^2H_2_O (Smith *et al.*, 2001) and [MnBr_2_(*bpym*)_2_]^.^CH_3_CN (Ha, 2011), have been investigated previously.

The asymmetric unit of the title compound, [MnI(*bpym*)_2_(H_2_O)]I^.^2H_2_O, contains a cationic Mn^II^ complex, an I^-^ anion and two solvate water molecules (Fig. 1). In the complex, the Mn^II^ ion is six-coordinated in a considerably distorted octahedral environment defined by four N atoms of the two chelating *bpym* ligands, one I^-^ anion and one O atom of a water ligand in a *cis*-N_4_IO coordination geometry. The main contribution to the distortion of the ocataheron is the tight N—Mn—N chelating angles (Table 1), which results in non-linear *trans* axes [<O1—Mn1—N1 = 167.23 (13)°, <I1—Mn1—N8 = 172.44 (9)° and <N4—Mn1—N5 = 158.58 (13)°]. The Mn—N(*bpym*) bond lengths are slightly different and longer than the Mn—O(H_2_O) bond (Table 1). Because of the different *trans* effects of the I and O atoms, the Mn1—N8 bond *trans* to the I atom is somewhat longer than the Mn1—N1 bond *trans* to the O atom. The dihedral angle between the least-squares planes of the two *bpym* ligands [maximum deviation = 0.088 (4) Å] is 76.48 (6)°. In the crystal structure, the complex, anion and solvate water molecules are linked by intermolecular O—H···O, O—H···I and O—H···N hydrogen bonds (Fig. 2, Table 2). In addition, the complexes display numerous inter- and intramolecular π-π interactions between adjacent pyrimidine rings, the shortest ring centroid-centroid distance being 3.611 (2) Å.

### Experimental

To a solution of 2,2'-bipyrimidine (0.1587 g, 1.003 mmol) in acetone (40 ml) was added MnI_2_ (0.1540 g, 0.499 mmol) and refluxed for 3 h. The formed precipitate was separated by filtration, washed with acetone and dried at 50 °C, to give a yellow powder (0.0701 g). Crystals suitable for X-ray analysis were obtained by slow evaporation from a methanol solution.

### Refinement

Carbon-bound H atoms were positioned geometrically and allowed to ride on their respective parent atoms [C—H = 0.95 Å and *U*_iso_(H) = 1.2*U*_eq_(C)]. The H atoms of the water ligand and solvent molecules were located from Fourier difference maps then allowed to ride on their parent O atoms in the final cycles of refinement with O—H = 0.84 Å and *U*_iso_(H) = 1.5 *U*_eq_(O). The highest peak (0.97 e Å^-3^) and the deepest hole (-1.15 e Å^-3^) in the difference Fourier map are located 1.38 Å and 0.85 Å from the I1 atom, respectively.

### Crystal data

**Table:**

[MnI(C_8_H_6_N_4_)_2_(H_2_O)]I·2H_2_O	*F*(000) = 1300
*M**_r_* = 679.12	*D*_x_ = 1.955 Mg m^−^^3^
Monoclinic, *P*2_1_/*c*	Mo *K*α radiation, λ = 0.71073 Å
Hall symbol: -P 2ybc	Cell parameters from 5481 reflections
*a* = 14.2105 (12) Å	θ = 2.4–28.0°
*b* = 21.5452 (19) Å	µ = 3.28 mm^−^^1^
*c* = 7.7064 (7) Å	*T* = 200 K
β = 102.063 (2)°	Stick, yellow
*V* = 2307.4 (4) Å^3^	0.25 × 0.23 × 0.11 mm
*Z* = 4	

### Data collection

**Table:**

Bruker SMART 1000 CCD diffractometer	5707 independent reflections
Radiation source: fine-focus sealed tube	3555 reflections with *I* > 2σ(*I*)
graphite	*R*_int_ = 0.049
φ and ω scans	θ_max_ = 28.3°, θ_min_ = 1.9°
Absorption correction: multi-scan (*SADABS*; Bruker, 2000)	*h* = −18→18
*T*_min_ = 0.838, *T*_max_ = 1.000	*k* = −26→28
17004 measured reflections	*l* = −10→10

### Refinement

**Table:**

Refinement on *F*^2^	Primary atom site location: structure-invariant direct methods
Least-squares matrix: full	Secondary atom site location: difference Fourier map
*R*[*F*^2^ > 2σ(*F*^2^)] = 0.037	Hydrogen site location: inferred from neighbouring sites
*wR*(*F*^2^) = 0.096	H-atom parameters constrained
*S* = 1.05	*w* = 1/[σ^2^(*F*_o_^2^) + (0.0334*P*)^2^] where *P* = (*F*_o_^2^ + 2*F*_c_^2^)/3
5707 reflections	(Δ/σ)_max_ < 0.001
271 parameters	Δρ_max_ = 0.97 e Å^−^^3^
0 restraints	Δρ_min_ = −1.15 e Å^−^^3^

### Special details

**Table:**

Geometry. All e.s.d.'s (except the e.s.d. in the dihedral angle between two l.s. planes) are estimated using the full covariance matrix. The cell e.s.d.'s are taken into account individually in the estimation of e.s.d.'s in distances, angles and torsion angles; correlations between e.s.d.'s in cell parameters are only used when they are defined by crystal symmetry. An approximate (isotropic) treatment of cell e.s.d.'s is used for estimating e.s.d.'s involving l.s. planes.
Refinement. Refinement of *F*^2^ against ALL reflections. The weighted *R*-factor *wR* and goodness of fit *S* are based on *F*^2^, conventional *R*-factors *R* are based on *F*, with *F* set to zero for negative *F*^2^. The threshold expression of *F*^2^ > σ(*F*^2^) is used only for calculating *R*-factors(gt) *etc*. and is not relevant to the choice of reflections for refinement. *R*-factors based on *F*^2^ are statistically about twice as large as those based on *F*, and *R*- factors based on ALL data will be even larger.

### Fractional atomic coordinates and isotropic or equivalent isotropic displacement parameters (Å^2^)

**Table:**

	*x*	*y*	*z*	*U*_iso_*/*U*_eq_	
Mn1	0.74435 (5)	0.08340 (3)	0.70450 (9)	0.02828 (17)	
I1	0.75344 (2)	0.159695 (15)	0.41319 (4)	0.03949 (11)	
O1	0.6323 (2)	0.02672 (15)	0.5586 (4)	0.0387 (8)	
H1A	0.5886	0.0426	0.4810	0.058*	
H1B	0.6080	−0.0013	0.6111	0.058*	
N1	0.8801 (2)	0.12304 (17)	0.8721 (5)	0.0308 (9)	
N2	1.0506 (3)	0.11088 (19)	0.9524 (5)	0.0384 (10)	
N3	1.0330 (2)	−0.00162 (18)	0.7835 (5)	0.0333 (9)	
N4	0.8656 (2)	0.01744 (17)	0.6830 (5)	0.0280 (8)	
N5	0.6390 (2)	0.13700 (17)	0.8319 (5)	0.0298 (9)	
N6	0.5241 (3)	0.12409 (18)	1.0153 (5)	0.0320 (9)	
N7	0.6065 (3)	0.01418 (18)	1.1326 (5)	0.0327 (9)	
N8	0.7162 (2)	0.02507 (17)	0.9400 (5)	0.0288 (8)	
C1	0.8881 (4)	0.1778 (2)	0.9580 (7)	0.0403 (13)	
H1	0.8314	0.2005	0.9628	0.048*	
C2	0.9750 (4)	0.2017 (2)	1.0380 (7)	0.0462 (13)	
H2	0.9801	0.2410	1.0946	0.055*	
C3	1.0552 (4)	0.1664 (2)	1.0338 (7)	0.0457 (14)	
H3	1.1164	0.1819	1.0907	0.055*	
C4	0.9630 (3)	0.0922 (2)	0.8738 (6)	0.0277 (10)	
C5	0.9540 (3)	0.0323 (2)	0.7750 (6)	0.0272 (10)	
C6	1.0229 (4)	−0.0540 (2)	0.6875 (6)	0.0376 (12)	
H6	1.0779	−0.0792	0.6893	0.045*	
C7	0.9364 (3)	−0.0724 (2)	0.5873 (6)	0.0360 (11)	
H7	0.9310	−0.1094	0.5188	0.043*	
C8	0.8576 (3)	−0.0356 (2)	0.5890 (6)	0.0333 (11)	
H8	0.7963	−0.0479	0.5226	0.040*	
C9	0.6009 (3)	0.1921 (2)	0.7805 (6)	0.0360 (11)	
H9	0.6273	0.2159	0.6985	0.043*	
C10	0.5239 (3)	0.2157 (2)	0.8434 (6)	0.0400 (12)	
H10	0.4980	0.2555	0.8088	0.048*	
C11	0.4864 (3)	0.1791 (2)	0.9574 (6)	0.0356 (12)	
H11	0.4313	0.1935	0.9973	0.043*	
C12	0.5992 (3)	0.1055 (2)	0.9501 (5)	0.0261 (10)	
C13	0.6433 (3)	0.0445 (2)	1.0125 (5)	0.0261 (10)	
C14	0.6486 (3)	−0.0404 (2)	1.1866 (6)	0.0338 (11)	
H14	0.6248	−0.0634	1.2735	0.041*	
C15	0.7240 (3)	−0.0644 (2)	1.1228 (6)	0.0356 (11)	
H15	0.7531	−0.1029	1.1639	0.043*	
C16	0.7557 (3)	−0.0294 (2)	0.9946 (6)	0.0355 (11)	
H16	0.8068	−0.0448	0.9447	0.043*	
I2	0.31079 (3)	0.174754 (18)	0.40131 (6)	0.05895 (14)	
O2	0.4688 (2)	0.06383 (16)	0.3226 (4)	0.0437 (9)	
H2A	0.4451	0.0977	0.3464	0.066*	
H2B	0.4931	0.0711	0.2343	0.066*	
O3	0.1581 (3)	0.3013 (2)	0.2003 (6)	0.0820 (14)	
H3A	0.1834	0.2718	0.2647	0.123*	
H3B	0.1906	0.2956	0.1222	0.123*	

### Atomic displacement parameters (Å^2^)

**Table:**

	*U*^11^	*U*^22^	*U*^33^	*U*^12^	*U*^13^	*U*^23^
Mn1	0.0226 (3)	0.0277 (4)	0.0337 (4)	0.0015 (3)	0.0041 (3)	0.0001 (3)
I1	0.0421 (2)	0.0334 (2)	0.0441 (2)	0.00048 (14)	0.01169 (16)	0.00641 (15)
O1	0.0323 (18)	0.043 (2)	0.0365 (19)	−0.0096 (15)	−0.0019 (15)	0.0068 (16)
N1	0.026 (2)	0.026 (2)	0.038 (2)	0.0035 (16)	0.0010 (18)	−0.0026 (17)
N2	0.025 (2)	0.033 (3)	0.054 (3)	−0.0005 (17)	−0.0002 (19)	−0.004 (2)
N3	0.028 (2)	0.031 (2)	0.041 (2)	0.0048 (17)	0.0071 (18)	0.0000 (19)
N4	0.026 (2)	0.026 (2)	0.031 (2)	0.0006 (16)	0.0035 (17)	0.0010 (16)
N5	0.027 (2)	0.030 (2)	0.033 (2)	0.0018 (16)	0.0060 (17)	0.0003 (17)
N6	0.030 (2)	0.033 (2)	0.034 (2)	0.0034 (17)	0.0086 (18)	−0.0019 (18)
N7	0.036 (2)	0.029 (2)	0.032 (2)	0.0006 (17)	0.0052 (18)	0.0009 (17)
N8	0.026 (2)	0.029 (2)	0.029 (2)	0.0038 (16)	0.0005 (17)	0.0021 (17)
C1	0.037 (3)	0.028 (3)	0.054 (3)	0.007 (2)	0.006 (3)	−0.007 (2)
C2	0.044 (3)	0.028 (3)	0.061 (4)	0.000 (2)	−0.001 (3)	−0.009 (3)
C3	0.034 (3)	0.034 (3)	0.063 (4)	−0.007 (2)	−0.001 (3)	−0.011 (3)
C4	0.026 (2)	0.028 (3)	0.030 (3)	0.0016 (19)	0.008 (2)	0.003 (2)
C5	0.026 (2)	0.024 (3)	0.032 (3)	0.0012 (18)	0.007 (2)	0.0037 (19)
C6	0.043 (3)	0.028 (3)	0.045 (3)	0.011 (2)	0.018 (3)	0.001 (2)
C7	0.042 (3)	0.027 (3)	0.041 (3)	0.000 (2)	0.014 (2)	−0.004 (2)
C8	0.035 (3)	0.031 (3)	0.034 (3)	−0.008 (2)	0.007 (2)	−0.002 (2)
C9	0.041 (3)	0.030 (3)	0.036 (3)	0.009 (2)	0.006 (2)	0.003 (2)
C10	0.043 (3)	0.029 (3)	0.047 (3)	0.016 (2)	0.008 (2)	0.003 (2)
C11	0.035 (3)	0.034 (3)	0.038 (3)	0.009 (2)	0.008 (2)	−0.002 (2)
C12	0.024 (2)	0.026 (3)	0.025 (2)	−0.0002 (18)	−0.0007 (19)	−0.0024 (19)
C13	0.023 (2)	0.027 (3)	0.027 (2)	−0.0007 (18)	0.0013 (19)	−0.0027 (19)
C14	0.037 (3)	0.033 (3)	0.029 (3)	−0.003 (2)	0.002 (2)	0.002 (2)
C15	0.038 (3)	0.029 (3)	0.036 (3)	0.004 (2)	0.001 (2)	0.006 (2)
C16	0.024 (2)	0.036 (3)	0.042 (3)	0.009 (2)	−0.002 (2)	−0.002 (2)
I2	0.0455 (2)	0.0452 (3)	0.0851 (3)	−0.00794 (17)	0.0113 (2)	0.0031 (2)
O2	0.039 (2)	0.051 (2)	0.044 (2)	−0.0021 (16)	0.0151 (17)	0.0064 (17)
O3	0.085 (3)	0.060 (3)	0.105 (4)	0.008 (2)	0.029 (3)	−0.004 (3)

### Geometric parameters (Å, °)

**Table:**

Mn1—O1	2.131 (3)	C1—H1	0.9500
Mn1—N1	2.253 (4)	C2—C3	1.375 (7)
Mn1—N4	2.266 (4)	C2—H2	0.9500
Mn1—N5	2.270 (4)	C3—H3	0.9500
Mn1—N8	2.310 (4)	C4—C5	1.489 (6)
Mn1—I1	2.8070 (8)	C6—C7	1.367 (6)
O1—H1A	0.8400	C6—H6	0.9500
O1—H1B	0.8400	C7—C8	1.375 (6)
N1—C1	1.346 (6)	C7—H7	0.9500
N1—C4	1.350 (5)	C8—H8	0.9500
N2—C4	1.329 (5)	C9—C10	1.383 (6)
N2—C3	1.346 (6)	C9—H9	0.9500
N3—C5	1.329 (5)	C10—C11	1.368 (7)
N3—C6	1.340 (6)	C10—H10	0.9500
N4—C8	1.345 (5)	C11—H11	0.9500
N4—C5	1.347 (5)	C12—C13	1.490 (6)
N5—C9	1.331 (6)	C14—C15	1.372 (6)
N5—C12	1.351 (5)	C14—H14	0.9500
N6—C12	1.334 (5)	C15—C16	1.390 (6)
N6—C11	1.339 (6)	C15—H15	0.9500
N7—C13	1.327 (5)	C16—H16	0.9500
N7—C14	1.344 (6)	O2—H2A	0.8400
N8—C16	1.332 (6)	O2—H2B	0.8400
N8—C13	1.343 (5)	O3—H3A	0.8400
C1—C2	1.361 (7)	O3—H3B	0.8400
			
O1—Mn1—N1	167.23 (13)	C2—C3—H3	118.6
O1—Mn1—N4	95.61 (13)	N2—C4—N1	126.0 (4)
N1—Mn1—N4	72.96 (13)	N2—C4—C5	117.9 (4)
O1—Mn1—N5	91.84 (13)	N1—C4—C5	116.1 (4)
N1—Mn1—N5	97.01 (13)	N3—C5—N4	125.5 (4)
N4—Mn1—N5	158.58 (13)	N3—C5—C4	118.1 (4)
O1—Mn1—N8	82.50 (12)	N4—C5—C4	116.5 (4)
N1—Mn1—N8	91.39 (13)	N3—C6—C7	122.4 (4)
N4—Mn1—N8	88.60 (13)	N3—C6—H6	118.8
N5—Mn1—N8	72.47 (13)	C7—C6—H6	118.8
O1—Mn1—I1	93.89 (9)	C6—C7—C8	117.8 (4)
N1—Mn1—I1	93.41 (10)	C6—C7—H7	121.1
N4—Mn1—I1	98.39 (9)	C8—C7—H7	121.1
N5—Mn1—I1	101.11 (10)	N4—C8—C7	121.2 (4)
N8—Mn1—I1	172.44 (9)	N4—C8—H8	119.4
Mn1—O1—H1A	120.1	C7—C8—H8	119.4
Mn1—O1—H1B	119.7	N5—C9—C10	121.7 (5)
H1A—O1—H1B	108.6	N5—C9—H9	119.2
C1—N1—C4	116.3 (4)	C10—C9—H9	119.2
C1—N1—Mn1	126.1 (3)	C11—C10—C9	117.2 (4)
C4—N1—Mn1	117.3 (3)	C11—C10—H10	121.4
C4—N2—C3	115.6 (4)	C9—C10—H10	121.4
C5—N3—C6	116.4 (4)	N6—C11—C10	122.8 (4)
C8—N4—C5	116.8 (4)	N6—C11—H11	118.6
C8—N4—Mn1	126.3 (3)	C10—C11—H11	118.6
C5—N4—Mn1	116.9 (3)	N6—C12—N5	125.7 (4)
C9—N5—C12	116.6 (4)	N6—C12—C13	117.3 (4)
C9—N5—Mn1	126.1 (3)	N5—C12—C13	117.0 (4)
C12—N5—Mn1	116.3 (3)	N7—C13—N8	125.9 (4)
C12—N6—C11	115.9 (4)	N7—C13—C12	117.4 (4)
C13—N7—C14	115.6 (4)	N8—C13—C12	116.7 (4)
C16—N8—C13	117.1 (4)	N7—C14—C15	123.4 (4)
C16—N8—Mn1	126.7 (3)	N7—C14—H14	118.3
C13—N8—Mn1	115.6 (3)	C15—C14—H14	118.3
N1—C1—C2	122.0 (4)	C14—C15—C16	116.3 (4)
N1—C1—H1	119.0	C14—C15—H15	121.9
C2—C1—H1	119.0	C16—C15—H15	121.9
C1—C2—C3	117.3 (5)	N8—C16—C15	121.7 (4)
C1—C2—H2	121.3	N8—C16—H16	119.1
C3—C2—H2	121.3	C15—C16—H16	119.1
N2—C3—C2	122.8 (5)	H2A—O2—H2B	105.5
N2—C3—H3	118.6	H3A—O3—H3B	94.7
			
O1—Mn1—N1—C1	−157.3 (5)	C1—N1—C4—N2	0.3 (7)
N4—Mn1—N1—C1	175.7 (4)	Mn1—N1—C4—N2	174.2 (3)
N5—Mn1—N1—C1	−23.7 (4)	C1—N1—C4—C5	−178.6 (4)
N8—Mn1—N1—C1	−96.2 (4)	Mn1—N1—C4—C5	−4.6 (5)
I1—Mn1—N1—C1	77.9 (4)	C6—N3—C5—N4	2.0 (6)
O1—Mn1—N1—C4	29.4 (8)	C6—N3—C5—C4	−177.5 (4)
N4—Mn1—N1—C4	2.4 (3)	C8—N4—C5—N3	−1.5 (6)
N5—Mn1—N1—C4	163.0 (3)	Mn1—N4—C5—N3	177.6 (3)
N8—Mn1—N1—C4	90.5 (3)	C8—N4—C5—C4	178.0 (4)
I1—Mn1—N1—C4	−95.4 (3)	Mn1—N4—C5—C4	−2.9 (5)
O1—Mn1—N4—C8	5.3 (4)	N2—C4—C5—N3	5.6 (6)
N1—Mn1—N4—C8	179.5 (4)	N1—C4—C5—N3	−175.5 (4)
N5—Mn1—N4—C8	115.1 (4)	N2—C4—C5—N4	−174.0 (4)
N8—Mn1—N4—C8	87.6 (4)	N1—C4—C5—N4	5.0 (6)
I1—Mn1—N4—C8	−89.5 (4)	C5—N3—C6—C7	−0.7 (7)
O1—Mn1—N4—C5	−173.8 (3)	N3—C6—C7—C8	−1.0 (7)
N1—Mn1—N4—C5	0.4 (3)	C5—N4—C8—C7	−0.3 (6)
N5—Mn1—N4—C5	−64.0 (5)	Mn1—N4—C8—C7	−179.3 (3)
N8—Mn1—N4—C5	−91.5 (3)	C6—C7—C8—N4	1.4 (7)
I1—Mn1—N4—C5	91.4 (3)	C12—N5—C9—C10	−0.7 (7)
O1—Mn1—N5—C9	−98.7 (4)	Mn1—N5—C9—C10	167.2 (4)
N1—Mn1—N5—C9	90.5 (4)	N5—C9—C10—C11	−1.7 (7)
N4—Mn1—N5—C9	150.8 (4)	C12—N6—C11—C10	−2.3 (7)
N8—Mn1—N5—C9	179.7 (4)	C9—C10—C11—N6	3.3 (7)
I1—Mn1—N5—C9	−4.4 (4)	C11—N6—C12—N5	−0.4 (6)
O1—Mn1—N5—C12	69.2 (3)	C11—N6—C12—C13	179.7 (4)
N1—Mn1—N5—C12	−101.6 (3)	C9—N5—C12—N6	1.8 (6)
N4—Mn1—N5—C12	−41.3 (5)	Mn1—N5—C12—N6	−167.3 (3)
N8—Mn1—N5—C12	−12.4 (3)	C9—N5—C12—C13	−178.3 (4)
I1—Mn1—N5—C12	163.5 (3)	Mn1—N5—C12—C13	12.7 (5)
O1—Mn1—N8—C16	87.0 (4)	C14—N7—C13—N8	1.3 (6)
N1—Mn1—N8—C16	−81.7 (4)	C14—N7—C13—C12	−179.5 (4)
N4—Mn1—N8—C16	−8.8 (4)	C16—N8—C13—N7	−0.6 (6)
N5—Mn1—N8—C16	−178.6 (4)	Mn1—N8—C13—N7	170.9 (3)
O1—Mn1—N8—C13	−83.5 (3)	C16—N8—C13—C12	−179.8 (4)
N1—Mn1—N8—C13	107.8 (3)	Mn1—N8—C13—C12	−8.3 (5)
N4—Mn1—N8—C13	−179.3 (3)	N6—C12—C13—N7	−2.1 (6)
N5—Mn1—N8—C13	10.9 (3)	N5—C12—C13—N7	178.0 (4)
C4—N1—C1—C2	1.7 (7)	N6—C12—C13—N8	177.2 (4)
Mn1—N1—C1—C2	−171.7 (4)	N5—C12—C13—N8	−2.7 (6)
N1—C1—C2—C3	−2.3 (8)	C13—N7—C14—C15	−0.6 (7)
C4—N2—C3—C2	0.7 (8)	N7—C14—C15—C16	−0.7 (7)
C1—C2—C3—N2	1.1 (9)	C13—N8—C16—C15	−0.9 (6)
C3—N2—C4—N1	−1.4 (7)	Mn1—N8—C16—C15	−171.2 (3)
C3—N2—C4—C5	177.4 (4)	C14—C15—C16—N8	1.4 (7)

### Hydrogen-bond geometry (Å, °)

**Table:**

*D*—H···*A*	*D*—H	H···*A*	*D*···*A*	*D*—H···*A*
O1—H1A···O2	0.84	1.93	2.753 (4)	166.
O1—H1B···O2^i^	0.84	1.87	2.693 (4)	166.
O2—H2A···I2	0.84	2.63	3.419 (3)	157.
O2—H2B···N6^ii^	0.84	2.16	2.948 (5)	157.
O2—H2B···N7^ii^	0.84	2.29	2.884 (5)	128.
O3—H3A···I2	0.84	2.82	3.624 (4)	161.
O3—H3B···I2^iii^	0.84	2.73	3.517 (4)	157.

Symmetry codes: (i) −*x*+1, −*y*, −*z*+1; (ii) *x*, *y*, *z*−1; (iii) *x*, −*y*+1/2, *z*−1/2.
